# Clinical significance of N-terminal natriuretic peptide combined with inflammatory factors, oxidative stress factors and blood lipid detection in elderly patients with Type-2 diabetes complicated with coronary heart disease

**DOI:** 10.12669/pjms.38.5.5513

**Published:** 2022

**Authors:** Xiao-min Meng, Shi-xin Kang, Jing Li, Hui-tao Zhang, Meng Li

**Affiliations:** 1 Xiao-min Meng, Department of Cardiovascular Medicine, Affiliated Hospital of Hebei University, Baoding 071000, Hebei, China; 2 Shi-xin Kang, Department of Cardiovascular Medicine, Affiliated Hospital of Hebei University, Baoding 071000, Hebei, China; 3 Jing Li, Department of Cardiovascular Medicine, Affiliated Hospital of Hebei University, Baoding 071000, Hebei, China; 4 Hui-tao Zhang, Department of Cardiovascular Medicine, Baoding First Central Hospital, Baoding 071000, Hebei, China; 5 Meng Li, Department of Cardiovascular Medicine, Affiliated Hospital of Hebei University, Baoding 071000, Hebei, China

**Keywords:** N-terminal natriuretic peptide, Inflammatory factor, Oxidative stress factor, Type-2 diabetes complicated with CHD

## Abstract

**Objectives:**

To observe the clinical significance of N-terminal natriuretic peptide combined with inflammatory factors, oxidative stress factors and blood lipid detection in elderly patients with Type-2 diabetes complicated with CHD (CHD), and provide a theoretical basis for the diagnosis and treatment of elderly patients with Type-2 diabetes complicated with CHD.

**Methods:**

A total of 40 patients with Type-2 diabetes complicated with CHD admitted to Affiliated Hospital of Hebei University from July 2019 and July 2020 were selected as the experimental group, and 40 patients with CHD who were hospitalized in our hospital during the same period without diabetes were selected as the control group. Venous blood was taken from all patients on morning and fasting basis, and their serum inflammatory factors as well as antioxidant molecules were examined respectively. Serum inflammatory factors include serum tumor necrosis factor α (TNF-A), interleukin-6 (IL-6), and C-reactive protein. Antioxidant molecules include antioxidant molecules superoxide dismutase (SOD), glutathione peroxidase (GSH-Px), total antioxidant capacity (TAC), catalase (CAT), glutathione reductase (GR), N-terminal natriuretic peptide (NT-proBNP), white blood cells (WBC), hemoglobin (HBG), albumin (ALB) and blood lipid levels. The differences of the above indexes between experimental group and control group were compared and analyzed.

**Results:**

The serum levels of TNF-a, CRP, and IL-6 in the experimental group were apparently higher than those in the control group, with a statistically significant difference (P=0.00); The levels of SOD, TAC and CAT in the experimental group were significantly lower than those in the control group, with a statistically significant difference (P=0.00); The level of NT-proBNP and WBC count in the experimental group were significantly higher than those in the control group, with a statistically significant difference (NT-proBNP, P=0.01; WBC, P=0.00). However, no statistically significant difference was observed in the levels of HBG and ALB between the two groups (P>0.05). The experimental group had significantly higher TC and TG levels than the control group, with statistically significant differences (TC, P=0.01; TG, P=0.02), but had a significantly lower HDL level than the control group, with a statistically significant difference (P=0.00).

**Conclusion:**

Elderly patients with Type-2 diabetes complicated with CHD showed systemic microinflammation, decreased antioxidant molecule content, as well as myocardial damage and abnormal lipid metabolism compared with patients with CHD alone. For this reason, attention should be paid to the above risk factors in clinical practice, and proactive prevention and treatment should be taken to reduce the probability of related complications.

## INTRODUCTION

In the wake of the changes in people’s lifestyles and the incessant improvement of dietary habits in recent years, diabetes has shown a trend of increasing incidence worldwide year by year, and the patients invaded by such a disease are becoming younger and younger, bringing great burden to families and society.^1^ Diabetes is a major risk factor for coronary atherosclerotic heart disease (CHD), and patients suffering from diabetes complicated with CHD have a relatively poor prognosis.^2^

Patients with diabetes have a high incidence and mortality of CHD due to various risk factors of cardiovascular disease such as abnormal lipid metabolism, micro-inflammatory state, insulin resistance, etc.^3^ According to statistics, approximately 70%-80% of patients with diabetes die from cardiovascular complications.^4^ Consequently, cardiovascular disease has turned into the most severe disease threatening the lives of patients with diabetes among all diseases. It has been shown in a study conducted by Fan et al.^5^ that the risk of death from cardiovascular disease in diabetic patients is 2 to 4 times higher than that in patients with cardiovascular disease alone. Diabetic patients complicated with CHD are characterized by high incidence, early onset age, and rapid disease progression.^6^ Studies have shown that^7^ coronary artery diseases in diabetic patients with CHD are more severe and diffuse than those in patients with CHD alone, presenting multiple diseases interwoven in multiple branches.^8^

## METHODS

A total of 40 patients with Type-2 diabetes complicated with CHD admitted to Affiliated Hospital of Hebei University from July 2019 and July 2020 were selected as the experimental group, and 40 patients with CHD who were hospitalized in Affiliated Hospital of Hebei University during the same period without diabetes were selected as the control group. Among them, there were 27 males and 13 females in the experimental group, aged 66-82 years with an average of 73.25±5.17 years, and 25 males and 15 females in the control group, aged 65-80 years with an average of 72.95±4.34 years. No significant difference was observed in the comparison of the general data between the two groups, which was comparable between the groups (Table-I).

**Table I:** Comparative analysis of general data between the two groups (χ¯±S) n=40.

Indexes	Experimental group	Control group	t/χ^2^	P
Age (years old)	73.25±5.17	72.93±4.32	0.29	0.77
Gender (Male, %)	27 (67.5%)	25 (62.5%)	0.22	0.64
BMI (kg/m^2^)	23.76±2.73	24.15±2.81	0.64	0.52
Hypertension (cases, %)	24 (60%)	21 (52.5%)	0.46	0.50
Smoking history (cases, %)	19 (47.5%)	16 (40%)	0.43	0.7
History of alcoholism (cases, %)	15 (37.5%)	17 (42.5%)	0.21	0.65
Degree of coronary stenosis	68.27±12.41	69.58±13.72	0.45	0.66
Coronary stent placement (cases, %)	11 (27.5%)	14 (35%)	0.52	0.47

P>0.05.

### Inclusion criteria:

- Patients aged ≥ 65 years;
- Patients who meet the diagnostic criteria for CHD^9^ and have been confirmed by coronary angiography;
- Patients who volunteered to participate in the study and signed informed consent;
- Patients without mental illness and able to cooperate to complete the study;
- Patients with complete clinical data.

### Exclusion Criteria:

- Patients with acute myocardial infarction, severe heart failure and arrhythmia;
- Patients with diseases that affected the study results, such as acute infection, malignant tumor, thyroid disease or autoimmune disease;
- Patients with severe liver and kidney dysfunction that cannot be satisfactorily corrected;
- Patients who have recently taken related drugs that affect the study, such as hormones and immunosuppressants.

### Ethical Approval:

The study was approved on September 22, 2021 by the Institutional Ethics Committee of Affiliated Hospital of Hebei University, and written informed consent was obtained from all participants.

### Laboratory Examination Indexes:

A total of 80 patients were included in the experimental group and the control group. Venous blood was taken from all patients on morning and fasting basis, and their serum inflammatory factors as well as antioxidant molecules were examined respectively. Serum inflammatory factors include serum tumor necrosis factor α (TNF-A), interleukin-6 (IL-6) (ELISA method, the kits used were provided by R&D Systems, Frence) and C-reactive protein (CRP) (transmission immunoturbidimetric, the kits used were provided by Roche). Antioxidant molecules include antioxidant molecules superoxide dismutase (SOD), glutathione peroxidase (GSH-Px), total antioxidant capacity (TAC), catalase (CAT), glutathione reductase (GR) (radioimmunoassay, the kits used were provided by Elabscience), N-terminal natriuretic peptide (NT-proBNP), white blood cells (WBC), hemoglobin (HBG), albumin (ALB) and blood lipid levels. The differences of the above indexes between experimental group and control group were compared and analyzed.

### Statistical Analysis:

All the data were statistically analyzed by SPSS 20.0 software, and the measurement data were expressed as (X̄±s). Two independent sample t-test was used for inter-group data analysis, and χ^2^ was adopted for rate comparison. P<0.05 indicates a statistically significant difference.

## RESULTS

The comparative analysis of serum inflammatory factors between the two group (Table-II) suggested, the levels of serum TNF-A, CRP and IL-6 in the experimental group were significantly higher than those in the control group, with statistically significant differences (P=0.00).

**Table II:** Comparative analysis of serum inflammatory factor indexes between the two groups (χ¯±S) n=40.

Group	TNF-a (ng/L)*	CRP (mg/L) *	IL-6 (ng/L) *
Experimental group	43.47±8.27	4.61±0.57	16.47±5.45
Control group	36.35±9.83	3.22±0.53	13.96±5.29
t	9.86	11.42	7.33
p	0.00	0.00	0.00

* P<0.05.

the comparative analysis of antioxidant molecules between the two groups (Table-III) suggested: the serum levels of SOD, TAC and CAT in the experimental group were significantly lower than those in the control group, with statistically significant differences (P=0.00). No significant difference can be seen in the GSH-Px and GR levels of the experimental group and the control group.

**Table III:** Comparative analysis of antioxidant molecules between the two group (U/ml, n=60,χ¯±S).

Group	SOD*	GSH-Px	TAC*	CAT*	GR
Experimental group	61.10±7.49	338.74±27.51	10.92±1.75	7.41±1.32	122.91±10.25
Control group	68.36±8.13	339.63±28.25	14.71±2.60	10.73±2.17	123.63±9.72
t	4.15	0.14	7.64	8.27	0.32
p	0.00	0.88	0.00	0.00	0.75

* P<0.05.

The comparative analysis of BNP and blood routine indexes between the two group (Table-IV) suggested: the serum level of NT-proBNP and WBC count in the experimental group were significantly higher than those in the control group, with a statistically significant difference (NT-proBNP, P=0.01; WBC, P=0.00). However, no statistically significant difference was observed in the levels of HBG and ALB between the two groups (P>0.05).

**Table IV:** Comparison and analysis of NT-proBNP and blood routine indexes between the two groups (χ¯±S) n=40.

Observation indexes	Experimental group	Control group	t	p
NT-proBNP (ng/L)*	63.71±6.38	60.21±5.72	2.58	0.01
WBC (×10^9^/L)*	6.98±1.37	5.57±2.01	3.05	0.00
HBG (g/L)	117.53±12.39	117.46±13.85	0.75	0.45
ALB (g/L)	37.28±3.12	37.39±3.53	0.58	0.64

* p<0.05.

The comparative analysis of blood lipid indexes between the two group (Table-V) further showed that the experimental group had significantly higher TC and TG levels than the control group, with statistically significant differences (TC, P=0.01; TG, P=0.02), but had a significantly lower HDL level than the control group, with a statistically significant difference (P=0.00). No significant difference was observed in other indexes between the two groups (P >0.05).

**Table V:** Comparative analysis of various lipid indexes between the two groups (χ¯±S) n=40.

Observation indexes	Experimental group	Control group	t	p
TC (mmol/L)*	8.53±1.76	7.62±1.25	2.67	0.01
TG (mmol/L)*	3.72±1.31	3.08±1.07	2.39	0.02
LDL (mmol/L)	5.34±1.30	5.35±1.27	0.03	0.79
VLDL (mmol/)	2.03±0.41	2.06±0.57	0.27	0.56
HDL (mmol/)*	1.18±0.22	1.37±0.26	3.53	0.00
ApoA (g/L)	1.46±0.93	1.45±0.72	0.05	0.95
ApoB (g/l)	1.16±0.21	1.18±0.32	0.33	0.74

* p<0.05.

## DISCUSSION

Diabetes is one of the principal chronic diseases endangering human health, and more than 90% of diabetic patients have Type-2 diabetes.^10^ In the wake of the incessant understanding of Type-2 diabetes and the improvement of various treatment methods, patients with ketoacidosis and infection have a significantly decreased mortality, and cardiovascular-related complications have turned into the most severe diseases threatening the lives of diabetic patients.^11^ It is currently recognized that Type-2 diabetes is an independent pathogenic factor of CHD.^12^ Studies have shown that^13^ most diabetic patients are at a high risk of future cardiovascular events, while cardiovascular patients with diabetes have a higher absolute risk of future cardiovascular events.

It has been shown in more and more studies that the pathogenesis of diabetic cardiovascular diseases has a close bearing on diabetic lipid metabolism disorders.^14^ Abnormal glucose metabolism can give rise to a series of adverse consequences, such as promoting the occurrence of coronary atherosclerosis, causing endothelial dysfunction, and ultimately leading to serious atherosclerotic diseases. A number of studies have proved that coronary artery diseases in diabetic patients with CHD are more diffuse and complex than those with CHD alone.^15^ Approximately 40.5%-50% of patients with Type-2 diabetes are complicated with abnormal lipid metabolism, mainly manifested as increased TG and TC, decreased HDC, as well as elevated or normal LDLC.^16^ According to Katsiki et al.^17^, diabetic patients with dyslipidemia characterized by low-density lipoprotein (LDL), moderately elevated cholesterol, and reduced high-density lipoprotein levels may suffer from an increased risk of CHD. It was considered in a study by Goodarzi et al.^18^ that a reduction of HDL-C by 10mg·dL may contribute to a 22% increase in the risk of CHD. It was confirmed in our study that the levels of TC and TG in elderly patients with Type-2 diabetes complicated with CHD were significantly higher than those of CHD alone, with a statistically significant difference (TC, P=0.01; TG, P=0.02). The HDL level was significantly lower than the control group, with a statistically significant difference (P=0.00). However, no significant difference was observed in serum lipids indexes such as VLDL, ApoA and ApoB7B between the two groups (P>0.05).

According to the study,^19^ diabetic patient have coronary artery disease that continues to progress rapidly even with optimized drug therapy. Inflammation-related indicators such as white blood cells, erythrocyte sedimentation rate, C-reactive protein and endothelin levels in diabetic patients are also significantly higher than those in non-diabetic patients. In addition, high inflammatory stress in diabetic patients is a key factor to promote the progression of atherosclerosis.^20^ It was considered by Martin et al.^21^ that changes in microinflammatory state, oxidative stress, glucagon-like peptide agonist compounds and intestinal microbiota increased the susceptibility to cardiovascular disease in Type-2 diabetes. According to the study by Frydrych et al.^22^, patients with Type-2 diabetes have immune system dysfunction, which in turn leads to chronic low-grade inflammation and oxidative stress dysfunction in the body, resulting in rapid progression of large vessel lesions including coronary artery. Akash et al. believed that inflammatory activation and coronary lesions in diabetic patients could be attributed to multiple factors^23^: glucolipid toxicity, production of reactive oxygen species (ROS), epigenetic factors, activation of various transcription-mediated pathways and various pro-inflammatory cytokines such as tumor necrosis factor-α (TNF-α), CRP and IL-6. It was confirmed in our study that the serum levels of TNF-a, CRP, IL-6 and WBC in the experimental group were significantly higher than those in the control group, with a statistically significant difference (P=0.00). The levels of SOD, TAC and CAT in the experimental group were significantly lower than those in the control group, with a statistically significant difference (P=0.00).

BNP was originally discovered from pig brain tissue by Sudon Equal in 1988 and named as brain natriuretic peptide. It is mainly produced in cardiomyocytes.^24^ Nt-probnp is of greater clinical significance by virtue of its larger molecules and richer peripheral blood content.^25^ Studies have shown that.^26^ the level of NT-proBNP in plasma is significantly positively correlated with the degree of coronary artery stenosis, and it is basically the same as the evaluation of Gensini coefficient, which reflects the condition of coronary artery disease.^27^ Moreover, with the increase of the number of diseased coronary arteries, the content of NT-probNP also increased.^28^ It was confirmed in the study by D’Amato et al.^29^ that the serum level of NT-proBNP in the CHD group was significantly higher than that in the non-CHD group. As the number of coronary artery diseases increased, the level of NT-probNP also increased. NT-proBNP is positively correlated with the degree of coronary artery disease, which contributes to the evaluation of the disease and the guidance of treatment. According to our results, the serum level of BNP in the experimental group was significantly higher than that in the control group, with a statistically significant difference (P=0.01), suggesting that the severity of coronary artery disease in patients with Type-2 diabetes complicated with CHD was higher than that in the CHD alone group.

### Limitation of the study:

A small sample size was included, only the laboratory examination data of elderly patients with Type-2 diabetes complicated with CHD and patients with CHD alone were compared, while the treatment and follow-up contents were not included. In response to this, active countermeasures will be taken to further expand the sample size, increase follow-up and treatment content, and confirm the value of the above indexes for the treatment effect and prognosis judgment, so that patients can effectively obtain various benefits in the treatment.

## CONCLUSIONS

Elderly patients with Type-2 diabetes complicated with CHD showed systemic microinflammation, decreased antioxidant molecule content, as well as myocardial damage and abnormal lipid metabolism compared with patients with CHD alone. For this reason, attention should be paid to the above risk factors in clinical practice, and proactive prevention and treatment should be taken to reduce the probability of related complications.

### Authors’ Contributions:

**XMM &** **SXK:** Designed this study, prepared this manuscript, are responsible and accountable for the accuracy and integrity of the work.

**JL &** **HTZ:** Collected and analyzed clinical data.

**ML:** Significantly revised this manuscript.
